# Randomized clinical trial of remote ischaemic preconditioning versus no preconditioning in the prevention of perioperative myocardial infarction during open surgery for ruptured abdominal aortic aneurysm

**DOI:** 10.1002/bjs5.55

**Published:** 2018-03-26

**Authors:** T. F. Pedersen, J. Budtz‐Lilly, C. N. Petersen, J. Hyldgaard, J.‐O. Schmidt, R. Kroijer, M.‐L. Grønholdt, N. Eldrup

**Affiliations:** ^1^ Department of Heart, Lung and Vascular Surgery Aarhus University Hospital Aarhus Denmark; ^2^ Department of Vascular Surgery Aalborg University Hospital Aalborg Denmark; ^3^ Department of Vascular Surgery Kolding Hospital Kolding Denmark

## Abstract

**Background:**

Remote ischaemic preconditioning (RIPC) has been suggested as a means of protecting vital organs from reperfusion injury during major vascular surgery. This study was designed to determine whether RIPC could reduce the incidence of perioperative myocardial infarction (MI) during open surgery for ruptured abdominal aortic aneurysm (AAA). Secondary aims were to see if RIPC could reduce 30‐day mortality, multiple organ failure, acute intestinal ischaemia, acute kidney injury and ischaemic stroke.

**Methods:**

This randomized, non‐blinded clinical trial was undertaken at three vascular surgery centres in Denmark. Patients who had open surgery for ruptured AAA were randomized to intervention with RIPC or control in a 1 : 1 ratio. Postoperative complications and deaths were registered, and ECG and blood samples were obtained daily during the hospital stay.

**Results:**

Of 200 patients randomized, 142 (72 RIPC, 70 controls) were included. There was no difference in rates of perioperative MI between the RIPC and control groups (36 versus 43 per cent respectively), or in rates of organ failure. However, in the per‐protocol analysis 30‐day mortality was significantly reduced in the RIPC group (odds ratio 0·46, 95 per cent c.i. 0·22 to 0·99; P = 0·048).

**Conclusion:**

RIPC did not reduce the incidence of perioperative MI in patients undergoing open surgery for ruptured AAA. Registration number: NCT00883363 ( http://www.clinicaltrials.gov).

## Introduction

Ruptured abdominal aortic aneurysm (AAA) is associated with high morbidity and mortality rates. Perioperative mortality ranges from 25 to 50 per cent, and rates of myocardial and intestinal ischaemia are high, in the region of 40 and 18 per cent respectively^1^, ^2^, ^3^.

Within the past 20 years there has been a focus on the importance of ischaemia–reperfusion injury following periods of low or completely abolished perfusion, particularly of the myocardium. Several studies^4^, ^5^, ^6^ have shown that, by inducing repeated transient ischaemia and subsequent reperfusion of an organ or extremity, it is possible to protect vital organs from ischaemia–reperfusion injury. This is the principle behind remote ischaemic preconditioning (RIPC).

Patients undergoing coronary bypass surgery with induced RIPC in an upper extremity had a total reduction in levels of troponin by 43 per cent when measured by area under the curve, reflecting reduced perioperative myocardial ischaemia^7^. The effects of RIPC in vascular surgery have been described previously^8^, ^9^, ^10^. One randomized study^11^ showed that RIPC of the lower extremities during elective aortic surgery reduced the myocardial infarction (MI) rate from 27 to 5 per cent, and demonstrated a significant reduction in renal damage.

Despite the significant interest in clinical effects of RIPC, few randomized trials have been conducted, and none solely on patients admitted for the treatment of ruptured AAA. This study therefore sought to determine whether RIPC during surgery for ruptured AAA could reduce the incidence of MI. Secondary endpoints were 30‐day mortality, and rates of multiple organ failure, intestinal ischaemia, acute kidney injury requiring dialysis, and ischaemic stroke.

## Methods

This randomized, non‐blinded clinical trial included patients with ruptured AAA. All patients were admitted to Aarhus University Hospital, Aalborg University Hospital or Kolding Hospital, Denmark, from April 2009 to December 2014. The study was conducted according to the CONSORT guidelines^12^ and registered at http://clinicaltrial.gov (NCT00883363). Informed consent was obtained after the operation, either from the patient or immediate relatives, according to ethical guidelines for acute studies in Denmark. The trial was performed in accordance with the Declaration of Helsinki, and the International Conference on Harmonization – Good Clinical Practice. The study was approved by the local research ethics committee.

### Randomization

All patients evaluated as capable of undergoing open repair for ruptured AAA were included and randomized in a 1 : 1 ratio to either preconditioning after commencement of anaesthesia or a control group. In a per‐protocol analysis, patients who died before vascular reconstruction were excluded from the analysis, as well as any patients who did not provide signed informed consent.

Randomization took place as soon as patient transfer or admission was made known, often before the patient was evaluated clinically by the vascular surgery staff. Randomization was performed by use of sealed envelopes with instructions regarding intervention. The randomization key was done by computer program with block randomization of 20 blocks of ten patients.

### Intervention

RIPC was carried out by using a conventional BP cuff as a tourniquet. This was placed on an upper arm of the patient and inflated to 50 mmHg above systolic BP, or at least 175 mmHg. The inflation period lasted 5 min and was repeated four times, with 5‐min interruptions between inflations. This intervention protocol for applying RIPC has been commonly used^13^. In the control group, the cuff was used in a conventional way simply for measurement of BP.

Aneurysm repair technique, anaesthesia and transfusion were at the discretion of the attending physicians, according to national and local hospital guidelines, and surgery was performed by vascular surgeons. The intervention started when the anaesthesia and surgical procedure allowed it. The goal was to initiate RIPC before the aortic clamp was set, and finish before the distal anastomosis was completed. RIPC was postponed until after induction of anaesthesia so that initial resuscitation or induction of anaesthesia was not compromised.

Preconditioning was defined according to the European Society of Cardiology^14^.

### Blinding

Interventions were initialized and carried out by the anaesthetic team, but were not blinded to the surgeon. Subsequent data collection was performed blinded. After all data had been recovered and validated, the database was locked and the randomization codes revealed.

### Data collection

During the operation and intensive care stay, all patients were monitored for myocardial ischaemia by real‐time, five‐lead continuous ECG and oxygen saturation. After the operation, and for the next 5 days, 12‐lead ECG was carried out and troponin T serum samples were collected daily. If the patient experienced chest pain or any other clinical symptoms of MI, an urgent ECG as well as troponin T sample were obtained, and repeated after 6 and 12 h.

### Outcomes

All complications were registered on case report forms during the hospital stay. After completion of the study, all journals were reviewed by a single author to ensure complete capture of all events that occurred during the study period. Dates of death were confirmed from the Danish national personal registry.

In accordance with the 2007 consensus definition of the European Society of Cardiology/American Heart Association^15^, MI was defined by a troponin T increase above the 99th percentile (corresponding to a level exceeding 50 ng/l) with either ECG changes compatible with ischaemia or the development of left bundle branch block and/or typical chest pain. MI was diagnosed during the hospital admission or during review of the clinical records, and then confirmed by two members of the trial team.

Secondary outcomes included: 30‐day mortality; multiple organ failure, defined as dysfunction of two or more organ systems; kidney injury, defined as a need for either temporary or permanent dialysis; stroke, defined as either ischaemic stroke or cerebral haemorrhage (all patients suspected of stroke underwent CT to exclude haemorrhage); and intestinal ischaemia, defined by surgical removal of ischaemic bowel.

### Statistical analysis

Sample size determination was based on previous publications related to randomized RIPC interventions. Ali and colleagues^11^ showed a reduction in the frequency of perioperative MI from 27 to 5 per cent (absolute risk reduction 22 per cent), corresponding to a relative risk reduction of 80 per cent. A conservative estimate placed the risk of MI at 30 per cent in the control group of the present study. To demonstrate a relative risk reduction of 50 per cent (incidence 15 per cent in the RIPC group) with 80 per cent power at a significance value of 0·05, 190 patients were deemed necessary, so 200 patients were planned for inclusion. Data were analysed on a per‐protocol basis. This was intentional, as the efficacy of preconditioning was hypothesized to attenuate the damage from reperfusion, thus requiring the patient to survive the vascular reconstruction. Therefore, patients who died before vascular reconstruction were excluded from the per‐protocol analysis. It was not hypothesized that RIPC can stop haemorrhage.

Patient characteristics and operative data were compared using the Mann–Whitney *U* test for continuous variables and the χ^2^ test for categorical variables. Categorical outcome data were analysed by means of Fisher's exact test. Odds ratios (ORs) were calculated by logistic regression and plotted on a logarithmic scale in a forest plot. Two‐sided *P <* 0·050 was considered statistically significant. SPSS® version 20.0 (IBM, Armonk, New York, USA) was used for statistical analyses.

## Results

**Figure 1:**
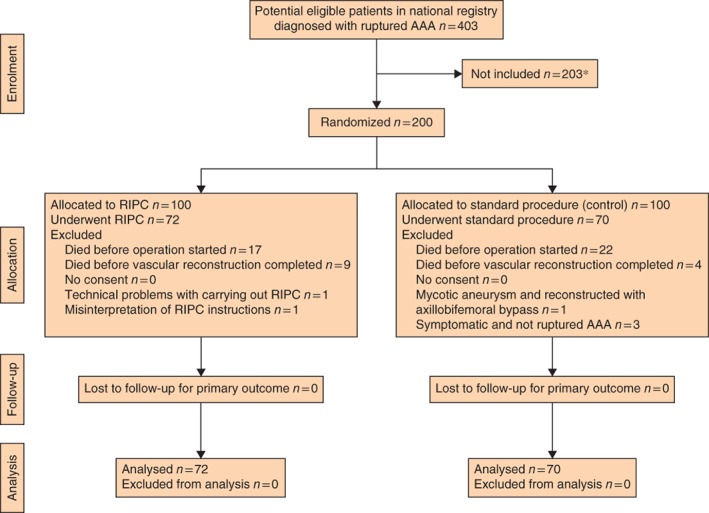
Flow chart for the study. *Mostly owing to problems with starting study protocol. AAA, abdominal aortic aneurysm; RIPC, remote ischaemic preconditioning

A total of 200 patients with ruptured AAA were randomized. Thirty‐nine patients died before surgery, and a further 13 died during the operation and were also excluded. A further six patients were excluded owing to protocol violations, leaving 142 for per‐protocol analysis (*Fig*. 1). This resulted in 72 patients being assigned to RIPC and 70 as controls. From internal data records at the participating centres, it was ascertained that 403 potentially eligible patients were treated between 2009 and 2014, of whom only 200 were included in the study.

Of the 142 patients included, 127 (89·4 per cent) were men. The mean(s.d.) age was 72·5(8·3) years. Baseline characteristics were similar between the two groups (Table 1). The previous medical record was unknown for six patients, three in each group.

**Table 1:** Participant characteristics

	RIPC group	Control group
	(n = 72)	(n = 70)
Age (years)*	72·3(8·6)	72·9(8·1)
Sex ratio (M : F)	63 : 9	64 : 6
BMI (kg/m^2^)†	26·7 (25·3, 28·0)	28·3 (27·2, 29·4)
Smoker		
Yes	39 (54)	30 (43)
No	24 (33)	29 (41)
Unknown	9 (13)	11 (16)
Previous medical record‡		
Ischaemic heart disease	20 (28)	15 (21)
Myocardial infarction	15 (21)	13 (19)
CABG/PCI	9 (13)	16 (23)
Stroke/TCI	8 (11)	9 (13)
Vascular surgery	3 (4)	2 (3)
Diabetes	5 (7)	8 (11)
Atrial fibrillation	10 (14)	11 (16)
COLD	11 (15)	12 (17)
Cancer	1 (1)	7 (10)
Hypertension	40 (56)	47 (67)
Preoperative medication		
Antiplatelet	29 (40)	23 (33)
Anticoagulant	3 (4)	3 (4)
Lipid‐lowering	29 (40)	28 (40)

Values in parentheses are percentages unless indicated otherwise; values are *mean(s.d.) and †mean (95 per cent c.i.).

‡ The previous medical record was unknown for six patients (3 in each group). RIPC, remote ischaemic preconditioning; CABG, coronary artery bypass grafting; PCI, percutaneous coronary intervention; TCI, transient cerebral ischaemia; COLD, chronic obstructive lung disease.

All patients had surgery under general anaesthesia. Mean(s.d.) duration of operation was 175(72) min. There were no significant differences between the two groups in procedure duration, red blood cell transfusions, and use of plasma and platelets (Table 2). There was no difference in type of graft (tube/bifurcated) used.

**Table 2:** Operative data

	RIPC group	Control group	
	(n = 72)	(n = 70)	P †
Duration of operation (min)	173(73)	175(70)	0·845
Autologous transfusion (ml)	993(984)	1439(1850)	0·427
Red blood cell transfusion (units)	8·7(6·7)	9·3(6·8)	0·628
Plasma (units)	5·5(3·3)	6·2(4·5)	0·354
Platelets (units)	1·8(1·3)	2·2(1·6)	0·203
Bifurcated prosthesis*	35 (49)	31 (44)	0·364‡

Values are mean(s.d.) unless indicated otherwise;

* values in parentheses are percentages. RIPC, remote ischaemic preconditioning.

† Mann–Whitney U test, except ‡χ^2^ test.

**Table 3:** Outcome by per‐protocol analysis

	RIPC group	Control group	
	(n = 72)	(n = 70)	P *
Myocardial infarction	26 (36)	30 (43)	0·502
30‐day mortality	14 (19)	24 (34)	0·035
Cause of death			
Cardiac	2	4	
Multiple organ failure	3	7	
Intestinal ischaemia	2	7	
Pulmonary	1	3	
Bleeding	4	1	
Renal	2	1	
Infection	0	1	
Multiple organ failure	10 (14)	11 (16)	0·472
Intestinal ischaemia	5 (7)	12 (17)	0·052
Dialysis	14 (19)	17 (24)	0·310
Stroke	2 (3)	2 (3)	0·679

Values in parentheses are percentages. RIPC, remote ischaemic preconditioning.

* Fisher's exact test.

### Primary outcome

**Figure 2:**
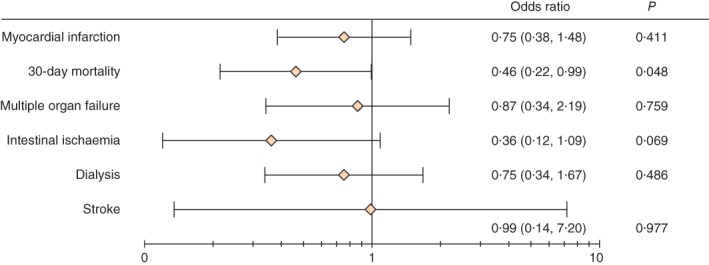
Forest plot showing effect of remote ischaemic preconditioning versus standard treatment on myocardial infarction and secondary endpoints. Odds ratios are shown with 95 per cent confidence intervals on a logarithmic scale for the per‐protocol analysis

There were 26 MIs (36 per cent) in the RIPC group and 30 (43 per cent) in the control group during the hospital admission (P = 0·502), an absolute difference of 7 per cent. The OR for RIPC versus control was 0·75 (95 per cent c.i. 0·38 to 1·48; P = 0·411) (Fig. 2).

### Secondary outcomes

There were 14 deaths (19 per cent) in the RIPC group and 24 (34 per cent) in the control group by 30 days (*P* = 0·035), an absolute risk reduction of 15 per cent. The OR for RIPC *versus* control was 0·46 (95 per cent c.i. 0·22 to 0·99; *P* = 0·048) by per‐protocol analysis. Multiple organ failure was identified in ten patients (14 per cent) in the RIPC group and 11 (16 per cent) in the control group (*P* = 0·472). Intestinal ischaemia developed in five patients (7 per cent) in the RIPC group and 12 (17 per cent) in the control group (*P* = 0·052). Acute kidney injury requiring dialysis occurred in 14 (19·4 per cent) and 17 (24 per cent) respectively (*P* = 0·310). There were two ischaemic strokes (3 per cent) in each group.

## Discussion

In this non‐blinded RCT of patients undergoing open repair for ruptured AAA, there was no significant difference in rates of perioperative MI between those who received RIPC and those who did not. The absolute risk reduction was 7 per cent in favour of RIPC. Previous randomized trials have focused on the effect of RIPC in patients treated electively for an intact AAA with conflicting results, reflecting study size and the fact that MI was not a primary endpoint^11^ ^16^, ^17^, ^18^, ^19^. The present analysis is the first to evaluate the effect of RIPC in the acute setting in patients with ruptured AAA.

In earlier studies, the method of MI diagnosis could also be questioned, although in a randomized study^20^, in which MI was defined by concomitant ECG changes and rise in troponin level, a non‐significant absolute reduction of 5 per cent (RIPC 8 per cent, control 13 per cent) in the incidence of MI was observed, a finding similar to that reported here. The findings from these two studies are much smaller than the expected 50 per cent relative risk reduction estimated from the study by Ali and colleagues^11^, even though the same American Heart Association definition of MI was used. In light of these observations, it is still unclear whether RIPC has any benefit in decreasing the incidence of MI among patients with AAA. The effects of RIPC are arguably more modest than the initial assumed 50 per cent relative risk reduction, so the present and previous studies may have insufficient sample sizes and be prone to type II error.

The absolute reduction in mortality risk at 30 days was 15 per cent, yielding an OR of 0·46 (95 per cent c.i. 0·22 to 0·99). Two other studies^11^ ^17^ found no differences in mortality, whereas a further study^18^ reported a 30‐day mortality rate of 13·5 per cent with RIPC versus 0 per cent in a control group. The differences in observed mortality between studies are not surprising, given that patients in the present study underwent treatment for ruptured aneurysms, whereas all the other trials included only patients with intact aneurysms undergoing elective surgery.

A recent review^10^ addressed other effects of RIPC, including its influence on acute kidney injury and renal impairment among patients undergoing elective AAA repair. One study^17^ that looked at protective renal effects as the primary outcome found that RIPC had no significant impact on kidney damage, when assessed by serum creatinine levels and urinary output, whereas another^11^ reported a significant reduction in peak serum creatinine levels. No preoperative serum creatinine levels were obtained for patients in the latter study owing to the acute nature of patient recruitment, and so it was not possible to differentiate acute from chronic renal insufficiency.

In the present study, intestinal ischaemia was also not significantly reduced by RIPC. In another study^16^, where intestinal ischaemia was evaluated by use of biomarkers, RIPC attenuated release of biomarkers of intestinal ischaemia–reperfusion injury, with significantly lower levels of endotoxin and a modest impact on the severity of intestinal injury.

Regarding stroke, the effect of RIPC has been poorly investigated. One study^17^ reported rates of 0 and 3 per cent in RIPC and control groups, and another^16^ encountered no strokes at all. The low incidence makes it difficult to draw any conclusions about the effect of RIPC on stroke, but it is interesting to note that these low numbers were similar to those in the present analysis.

The present study has a number of limitations. The issue of exclusion is important, as 58 (29·0 per cent) of the randomized patients were excluded from the projected goal of 200, affecting the power of the study. A dropout rate of only 10 per cent was assumed, which in retrospect was too low. In addition, the power calculation was based on a 50 per cent relative risk reduction for MI. However, there turned out to be a much smaller reduction than anticipated, so that no significant difference would have been detected even if 100 patients survived in each arm. Based on the present finding of a 7 per cent absolute risk reduction in MI from 43 to 36 per cent, the trial would have required a sample size of approximately 1200 patients. Of 403 potentially eligible patients, only 200 were included in the study. There were two reasons for this. The study did not commence at the same time at all three centres, and there was some reluctance at the start on the part of surgeons and anaesthetists to accommodate an experimental intervention in an acute procedure.

The fact that this study was based in an acute setting also led to some limitations in baseline measurements. The RIPC treatment was initialized during surgery, similar to methods used in many elective studies, but the first set of variables was collected immediately after operation. Much of the focus of therapeutic conditioning has been on the preischaemic stage for tissue protection, but recent studies^21^, ^22^, ^23^, ^24^ have shown that postconditioning may also play an important role, thus questioning the role of timing in RIPC intervention. The acute setting may be an ideal target for RIPC, but the varying and often extreme decompensation of patients with ruptured AAA may have limited, if any, potential gain from conditioning.

The type of anaesthesia was not standardized, which could interfere with RIPC. A recent meta‐analysis^25^ has shown that some types of anaesthesia may protect against ischaemia–reperfusion injury. The standard acute anaesthesia protocol in Denmark at all three participating centres during the study period consisted of a propofol and fentanyl‐based anaesthesia, sometimes supported with sevoflurane, at the discretion of the anaesthetist.

The lack of blinding of the surgeon is another limitation that could have influenced outcomes, but was presumed not to because the operating surgeon was not involved in data collection, and the analysis was conducted unblinded. A sham intervention and blinding of the surgeon would have potentially limited the risk of confounding. Indeed, Li and colleagues^16^ and Mouton *et al*.^19^ blinded surgeons to the randomization in their studies, albeit in an elective setting.

The inclusion of non‐consecutive patients and the risk of selection bias are more concerning. Attempts were made to overcome unequal inclusion among the three vascular centres by using block randomization. The comparable patient demographics suggest, at any rate, that patient groups were similar.

Some medications could also have affected the results. Sulphonylureas and nicorandil affect mitochondrial function. Sulphonylurea is not used in Denmark and nicorandil only rarely, so it is unlikely that these drugs could have affected the main result.

This RCT showed that, despite the high incidence of MI, RIPC intervention had no significant effect, with an absolute risk reduction of only 7 per cent. RIPC failed to influence the incidence of multiple organ failure, acute kidney injury, intestinal ischaemia and stroke, but there was a statistically significant reduction in 30‐day mortality. No causal link with RIPC is apparent for this last finding.
